# Altered social cognition in a community sample of women with disordered eating behaviours: a multi-method approach

**DOI:** 10.1038/s41598-021-94117-4

**Published:** 2021-07-19

**Authors:** Devon S. Heath, Nimrit Jhinjar, Dana A. Hayward

**Affiliations:** 1 Department of Educational Psychology, University of Alberta, Edmonton, Canada; 2 Department of Psychology, P-217 Biological Sciences Building, University of Alberta, Edmonton, AB T6G 2R3 Canada; 3 Women & Children’s Health Research Institute, University of Alberta, 5-083 Edmonton Clinic Health Academy (ECHA), 11405 87 Avenue NW, Edmonton, AB T6G 1C9 Canada; 4 Neuroscience and Mental Health Institute, University of Alberta, 2-132 Li Ka Shing, Edmonton, AB T6G 2E1 Canada

**Keywords:** Psychology, Human behaviour

## Abstract

Prior work suggests that individuals with an eating disorder demonstrate task-based and overall differences in sociocognitive functioning. However, the majority of studies assessed specifically anorexia nervosa and often employed a single experimental paradigm, providing a piecemeal understanding of the applicability of various lab tasks in denoting meaningful differences across diverse individuals. The current study was designed to address these outstanding issues. Participants were undergraduate females who self-identified as having an official (n = 18) eating disorder diagnosis or disordered eating behaviours with no diagnosis (n = 18), along with a control group (n = 32). Participants completed three social tasks of increasing complexity with different outcome measures, namely a gaze cueing task, passive video-watching using eyetracking, and a task to measure preferred social distance. Results diverged as a function of group across tasks; only the control group produced typical social attention effects, the disordered eating group looked significantly more at faces, and the eating disorder group demonstrated a significantly larger preferred social distance. These results suggest variations in task efficacy and demonstrate that altered sociocognitive functioning extends beyond official eating disorder diagnosis.

## Introduction

Social cognition, or the act of perceiving, attending, remembering, and making judgments about social content, is a crucial process that allows us to navigate seamlessly through our environment^1,2^. In contrast, individuals with autism spectrum disorder (ASD)^3^, borderline personality disorder^4^, social anxiety^5^, and eating disorders such as anorexia nervosa (AN)^6^ and bulimia nervosa (BN)^7^ seem to show difficulties with various aspects of social cognition. For instance, those with an eating disorder have been found to have challenges with theory of mind^8^, emotional functioning^8^, and visual processing of body-related stimuli^8,9^. While there have been lab-based investigations focusing on differences in social cognition between different groups, this approach assumes that the various tasks used to measure social cognition are themselves effective at identifying meaningful differences in social cognition. Furthermore, and exacerbating this assumption, other potential limitations for determining the effectiveness of particular tasks do arise. For example, many investigations employ only one task to provide a measure of social cognition, which does not allow for meaningful cross-task comparisons or a determination of how representative that task is at providing an accurate measure of one’s social cognition. Also, many participant samples consist of highly homogenous subgroups^8–11^, which may minimize within-group differences and could lead to overly simplistic conclusions. Thus, our investigation's main aim was to (i) present multiple sociocognitive tasks, increasing in complexity, and (ii) employ a heterogenous, community sample of women who self-report various eating disorder behaviours. This was done to ascertain which tasks, if any, could stratify performance based on the way we grouped participants, namely those who self-reported receiving an official eating disorder diagnosis, those who self-reported eating disorder behaviours but no official diagnosis, or controls.

### Social cognition in eating disorders

Previous attempts to outline altered social cognition in individuals with an eating disorder tended to focus on executive function impairments such as theory of mind^12,13^ and emotional processing^14,15^. For instance, the data suggest that individuals with AN have difficulty deciphering mental states when looking at eyes or facial expressions of various cartoons^16^, demonstrating impacted theory of mind. Another area of focus when investigating social cognition is how individuals look at or attend to images of food or bodies^17,18^. For example, individuals with AN have been found to look at images of food for less time than controls^17^ and demonstrate early preferential looking towards specific body areas they deem unattractive^18^. While recent literature has shown that individuals with eating disorders have challenges with social cognition, these conclusions were based on a subset of sociocognitive measures, with a large focus on those diagnosed with AN. Thus our work is focused on assessing other sociocognitive measures across individuals with different eating disorder presentations.

### Task rationale

There are many ways to measure social cognition in the lab, from looking at or paying attention to a single item on-screen, all the way up to interacting with other people and their points of view (i.e., theory of mind). With that in mind, we chose tasks and activities that produce performance differences between neurotypical populations and those with reduced sociocognitive functioning. Ultimately, we sought to collect measures commonly employed in the field and assess different facets of social cognition.

First, we used the gaze cueing task because it is a standard laboratory tool in cognitive science, and there is some evidence that one’s level of real-world social competence is linked to performance on this task^19–21^. Second, we used eyetracking technology during a video-watching task to observe social attention during complex social scenarios with multiple stimuli as, currently, gaze behaviour is the most common measurement technique for studying social cognition in real-world settings. Finally, to increase ecological validity, we used the preferred interpersonal space task to measure one’s preferred interpersonal space in a social context.

### The cueing task

The cueing task is one of the most widely-used laboratory tasks to measure spatial attention^22,23^. Several researchers have used variations of this task to measure attention across the lifespan^24–26^, within various clinical populations^19,27,28^, and in combination with eyetracking^29,30^, EEG^31^, and fMRI^32,33^. The general procedure begins with a cue to direct attention to a spatial location, followed sometime later by a target that appears either in the cued or uncued location. By manipulating the type of spatial cue (i.e., a simple luminance change, symbolic shape, arrow, or face with an averted gaze), one can assess the cues' propensity to shift attention. Further, by manipulating the length of time between the presentation of the cue and the target, one can measure how quickly different cues shift attention, and by manipulating the likelihood that the cue predicts the location of the target (rarely, at chance, most of the time)^34,35^, one can again make claims about how automatically the various cues shift attention.

Turning to the gaze cueing task, which provides a measure of social attention^25,34,36,37^, control participants respond faster for gazed-at (cued) as compared to not gazed-at (uncued) targets, even when the face is unlikely to look at the target; this difference in response times is known as the gaze cueing effect. While this task assesses one’s natural propensity to orient attention in the direction of another’s gaze, individual differences have been found to modulate the magnitude of the gaze cueing effect. Specifically, individuals with anxiety^19^, ASD^21,27,38,39^, and those with a smaller social network size^38^ produce smaller gaze cueing effects, suggesting that the gaze cueing task may be a strong index of social functioning in real life.

There has been one investigation to date within the eating disorder population where individuals with AN completed three cueing tasks, with averted gaze, arrow, and a finger pointing left or right acting as cues^10^. Unlike controls, they found that the individuals with AN did not show early attention shifts for either the gaze or arrow cues; all three cues produced late shifts of attention for both groups. The authors took the lack of an early cueing effect as evidence for impaired bottom-up attention for gaze and arrow cues, but not for body-related cues^10^, suggesting that individuals with AN may not have a natural propensity to attend to eyes. While this data provides preliminary evidence that individuals with AN may not preferentially attend to eyes, there was no distinction from arrow cues, making it less clear whether it is attention in general, or social attention in particular, that is affected. Further, it is unclear whether absent early orienting for gaze is specific to those with AN or whether this pattern is a hallmark for those with an eating disorder in general. Finally, it remains unclear whether the cueing task parameters are adequate to detect differences in real-life social cognition.

### Video watching tasks

One way to increase the complexity of social content is to present various scenes or film clips to participants and ask them to look at, respond to, or judge what is happening^40,41^. Eyetracking technology is one way to observe naturalistic eye movements when passively viewing social stimuli^42^. Presenting complex social scenes to participants allows researchers to measure an individual’s propensity to look at certain stimuli over others, which is a limitation of the cueing task^43^. Moreover, while gaze behaviour may not fully index one’s attentional focus at all times^44^, eye movements are an important way to take in information and signal information to others. These so-called dual functions of gaze^45,46^ highlight the rich information one can attain through investigations into participant gaze behaviour while mimicking more ecologically-valid contexts than the cueing task, providing further insight into individual sociocognitive functions.

Research findings indicate that control individuals tend to look primarily at faces and eyes during social viewing tasks^47–49^. Similar to the individual variability found with the gaze cueing task, looking behaviour also varies across individuals, as participants with a higher number of autism-like traits or with social anxiety tend to avoid fixating on faces^50,51^. Intriguingly, some data suggest an optimal “looking rate” towards faces; those who focus less may miss important social cues, while those who over-fixate on faces may seem threatening during social interactions^52^. Turning to eyetracking for those with an eating disorder, only a handful of studies have investigated looking patterns for social stimuli. Pertinent to the current study, Pinhas and colleagues^9^ presented images of people with different body sizes and pictures of social interactions to those with and without AN. While control individuals spent similar amounts of time looking at all three types of images, those with AN looked most at thin body shapes, followed by larger body shapes, and looked least at pictures of social interactions, leading the authors to suggest that social interactions may not be as rewarding for those with AN^9^. To date, investigations into BN have been limited, focusing more on emotional processing rather than social interactions as a whole^14,15,53^. The closest study we could find measured the ability of patients with BN to “read the mind in the films”; while overall performance was similar between individuals with BN and controls, those with BN were better able to recognize negative emotions when watching films, potentially suggesting a bias for negative content^54^. Dovetailing with laboratory findings, real-time social interactions also provide evidence for reduced fixations on the eyes of a conversation partner for those diagnosed with AN^9,55^. Taken together, there is some research suggesting differences in gaze behaviour towards social content across those with and without AN; it remains unclear whether looking rates diverge within different manifestations of eating disorders.

### Preferred interpersonal space task

While laboratory tasks like the cueing task and passive viewing tasks allow for precise experimental control and measurement, it is also essential to consider how social cognition manifests in real-time settings^20,56^. One ecologically-valid way to measure social cognition in a face-to-face setting while still maintaining a degree of experimental control is by measuring preferred interpersonal space via the stop-distance paradigm^4^. The stop-distance paradigm is a live, social interaction in which a confederate walks towards a participant. The participant is instructed to say “stop” when they feel uncomfortable, therefore providing a quantifiable measure of one’s personal space. Social distance is the average preferred interpersonal space someone may exhibit around a stranger in the real world. The social distance someone chooses can represent appropriateness, comfort, and intimacy with another person^57^, impacting sociality.

The stop-distance paradigm has been used to provide a real-world measure of individual differences in sociality in those with borderline personality disorder^4^, social anxiety^5^, and ASD^58,59^. However, to date, this paradigm has not been employed to investigate preferred interpersonal space in those with an eating disorder. Those with borderline personality disorder and social anxiety have been found to have a considerably larger preferred social distance than controls. However, for those with ASD, the findings are mixed; while one study found that people with ASD present with a smaller preferred social distance than controls^58^, Perry and colleagues^5^ suggest that the preferred social distance in the ASD group is much more variable, with the level of social anxiety as a good predictor of distance. Despite potential discrepancies with the ASD findings, the stop-distance paradigm appears to be a useful tool to capture individual differences in social cognition in a real-world setting. As environmental context could influence one’s sociality, obtaining an interpersonal, dyadic measurement of social cognition provides another method of determining sociocognitive functioning for those with an eating disorder.

In sum, there is compelling evidence to suggest that individuals with eating disorders may have reduced sociocognitive ability, potentially comparable to individuals with ASD^10,27,56,58,59^. The dearth of information about how the broader eating disorder community perceives, attends, and interacts with the social world around them is a key reason to investigate this population. Moreover, there is a need to investigate the efficacy of various sociocognitive measures at highlighting potential differences in functioning across the spectrum of eating disorders.

The present study employed three tasks to obtain a multifaceted measure of sociocognitive function. The participant sample consisted of women who self-identified as currently experiencing or having a history of an eating disorder (ED), along with women with no formal diagnosis but who self-report having disordered eating habits (DE) and control individuals. In the lab, participants (i) performed a gaze cueing task to measure social attention to a single face, (ii) watched a short movie clip depicting complex social interactions while their eye movements were tracked, and (iii) completed a face-to-face task to measure preferred social distance in real life. If the tasks are sensitive enough to variations in sociocognitive function, then we would expect differences in performance across all three groups across all three measures. If, however, some tasks are more sensitive than others at detecting variations in social cognition, then only some measures will indicate a meaningful difference between the ED and DE groups.

## Method

### Participants

A total of 72 undergraduate female students participated in the study, for which 68 were retained; 32 controls (M = 19.2 years, *SD* = 1.39) and 36 participants who identified as either currently (n = 16) or at some point in the past (n = 20) having an eating disorder (M = 19.7 years, *SD* = 1.65; see Table 1 for further details). Prior to data collection, participants completed a general pre-screening questionnaire, where they were asked, “Have you ever been diagnosed with an eating disorder?”. Those individuals who answered NO were eligible to participate as a control participant, and those individuals who answered YES and signed up for the study completed a lab-generated questionnaire, where those individuals who reported having an official diagnosis were classified as the eating disorder group (n = 18, M = 19.1, *SD* = 0.87; henceforth denoted ED; n = 8 currently experiencing ED, n = 10 remitted/recovered ED) and those 18 participants who self-reported experiencing eating disorder symptoms with no formal diagnosis were classified as the disordered eating group (M = 20.3, *SD* = 2; henceforth denoted DE; n = 8 currently experiencing ED, n = 10 remitted/recovered ED). We did not calculate Body Mass Index (BMI) due to unreliable weight data and the lack of height information. Instead, we relied on a combination of validated ED psychopathology questionnaires and self-reported ED-relevant information. We performed post-hoc independent sample t-tests between the three groups on the eating disorder examination questionnaire short (EDE-QS) scores. The control group scored significantly lower than the ED (*t*(48) = − 5.6, *p* < 0.001) and DE groups (*t*(48) = − 6.8, *p* < 0.001), while there was no difference between the ED and DE group (t(34) = 2.0, *p* > 0.05).

**Table 1:** Depicts participant information, including sample size, mean age (SD), mean Eating Disorder Examination Questionnaire short scores (EDE-QS; SD), and eating disorder diagnosis for the ED group.

	Control	DE	ED
Total number of participants	32	18	18
Mean age (years; *SD*)	19.2 (*1.39*)	20.3 (*.87*)	19.7 (*1.65*)
EDE-QS	5.7 (*6.48*)	21.2 (*9.54*)	15.94 (*5.5*)
Anorexia nervosa	–	–	9
Bulimia nervosa	–	–	3
Binge-eating disorder	–	–	3
Avoidant/restrictive food intake disorder	–	–	1
AN/BED	–	–	1
AN/BN	–	–	1

Sample size was determined in the following manner. For the gaze cueing task, we referred to a paper that compiled and calculated sample sizes from the literature, where they found that employing a detection task with fixation cue required a sample size of 10 participants^60^. Turning to the video-watching and stop-distance tasks, less prior work has been conducted, rendering it more difficult to ascertain an adequate sample size. We thus used G*Power 3.1^61^ to estimate the sample size needed to detect differences (if any) between the three groups in the proportion of time spent looking at the eyes or preferred interpersonal distance via repeated-measures ANOVAs with a between-subjects factor. We estimated an effect size *f* = 0.25, α = 0.05, Power = 0.9, three groups, and two measures, and returned a total sample size of 54 participants.

Participants were all recruited through the University of Alberta’s introductory psychology research participant pool and compensated with course credit. All procedures and protocols were reviewed and approved by the University of Alberta’s Research Ethics Board 2 (REB 2) and adhere to the principles of the Helsinki Declaration. All participants provided written informed consent.

### Apparatus

The gaze cueing task and video watching task were run on a Windows PC using a 22.5-in VIEWPixx monitor. Matlab and Psychtoolbox-3^62^ were used to run the gaze cueing task, while Experiment Builder (SR Research; Mississauga, ON) was used to run the video watching task. Eye movements were recorded using the EyeLink 1000 Plus eye tracker (SR Research; Mississauga, ON), with a temporal resolution of 2000 Hz and a spatial resolution of at least 1˚. Nine-point calibration and drift correction were performed at the beginning of the task and repeated during the experiment as necessary (the average spatial error was no greater than 0.42, with maximum error not exceeding 0.80). DataViewer (SR Research, Mississauga, ON) was used to visualize the gaze behaviours and extract the values.

### Stimuli

For the gaze cueing task, the stimuli were similar to prior studies^34,39,63^ that utilized a picture of a female face (6.5 cm W × 8.6 cm H; roughly 4.7 × 6.2 degrees of visual angle) depicting a neutral expression taken from the B series of the Karolinska Directed Emotional Faces database (KDEF)^64^, model code BF23NES. Eye gaze was adjusted to look left or right with Adobe Photoshop software. The target “X” (approximately 0.6 × 0.7 degrees of visual angle) appeared 6.4 degrees of visual angle (°) to the left or right of the face on-screen. Participants sat approximately 70 to 90 cm from the screen with their head on a chin rest.

For the video watching task, participants watched a six-minute and 15-s video clip of the American version of The Office (NBC), at dimensions of 12.7° (H) × 22° (W), centred on a grey screen. The clip was a shortened version of the Pilot episode of The Office, chosen to include both social and non-social scenes. The included segments from the episode were 0:00–3:00 min, 3:30–4:16 min, 8:39–8:52 min, 8:57–9:17 min 14:00–15:30 min, and 22:13–22:28 min. The clips were cut by exporting the episode from a DVD and using iMovie.

### Design and procedure

The tasks were completed as part of a larger ongoing study, whereby participants filled out a battery of self-reports, completed a gaze cueing task, a video watching task with a passive watching and event segmentation component, and a preferred interpersonal space task. The present investigation concentrated on analyzing the gaze cueing task, passive watching, and preferred interpersonal space task. We chose not to include the event segmentation component for the video watching task, whereby individuals made button presses to signify what they perceived to be separate events within the video, as this task addresses a different research question.

#### Gaze cueing task

**Figure 1:**
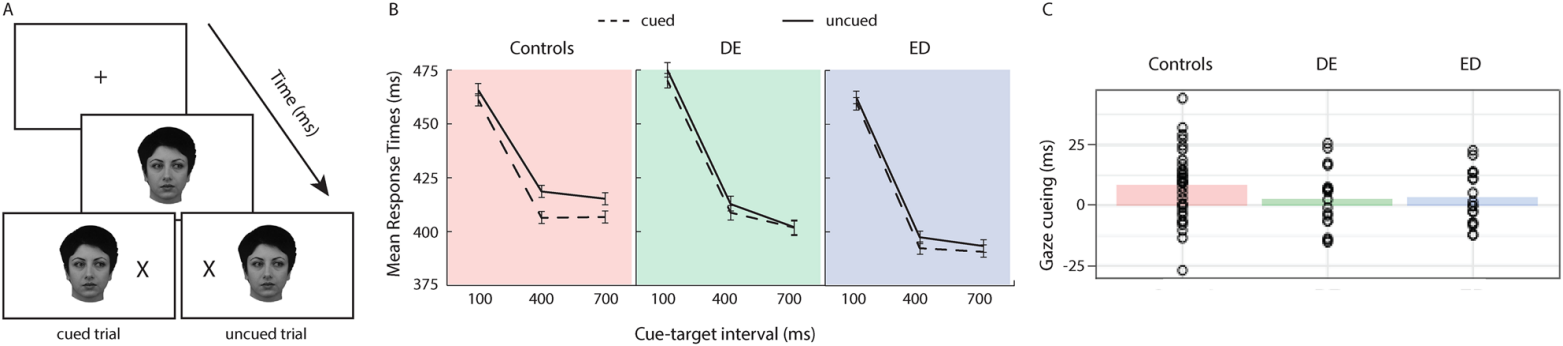
(**A**) depicts the gaze cueing task sequence, with the bottom-left image depicting a cued trial and the bottom-right depicting an uncued trial. (**B**) shows mean interparticipant correct response times as a function of *Cue validity* and *Cue-target interval* by group (Controls, DE, and ED; error bars are standard error of the difference between the means). (**C**) depicts both the average magnitude of gaze cueing (calculated as the average uncued RT—the average cued RT) for each group (coloured bars) and the individual data points illustrating each participant’s magnitude of gaze cueing.

Standard procedures were used for the gaze cueing task^25,63^. Gaze direction (left, right), target location (left, right), and cue-target interval (100, 400, 700 ms) were all equally likely to occur, and participants were informed of this contingency. As per Fig. 1A, each trial began with a fixation cross in the middle of the screen, where participants were asked to focus their attention for the duration of the task. Next, the female face appeared in the center of the screen with eyes looking either left or right. After a cue-target interval of 100, 400, or 700 ms, the target appeared on either the left or right and participants were asked to detect the target via keypress as quickly and as accurately as possible once they perceived the target. The cue and the target remained on the screen until a response was made or until 1,000 ms had passed. No error feedback was provided. The eye tracker was recalibrated between blocks as needed.

Participants completed 228 trials split across six blocks of 38 trials, with 36 target-present trials and two target-absent trials per block. The target-absent, or ‘catch’ trials, were to ensure participants were paying attention and not responding sporadically, and accounted for roughly 6% of all trials. Response time was measured from target onset, and participants completed five practice trials at the start of the task.

#### Video watching task

Participant eye movements were tracked with the eye tracker for the duration of the video. Before the video started, nine-point calibration was completed. Participants passively watched a 6-min and 15-s clip from the American version of The Office (NBC), and were simply instructed to “watch the video”. The Office was chosen to maintain consistency with pre-existing research^65^ and due to its fast-paced structure and complicated social nature.

#### Preferred interpersonal space task

Participants began standing face-to-face, 9 feet, 5 inches away from one of three female confederates (who strove to maintain consistency in eye contact, gait, posture, and clothing across all participants). The confederate slowly approached the participant, who was instructed to “say stop when you feel uncomfortable”^4^. Final toe-to-toe and nose-to-nose distance were measured using a measuring tape, save for the first two individuals we tested, where we obtained just the toe-to-toe distance. Thus those two individuals were removed from the relevant analyses. A total of three trials were run, and mean preferred distances (cm) were calculated.

#### Eating disorder examination questionnaire short (EDE-QS)

The EDE-QS is a validated twelve-question self-report tool that measures the range and severity of eating disorder features^66,67^. Participants answer a set of statements regarding their behaviours and attitudes. The first ten questions investigate the frequency of disordered eating within the last seven days. Subscales also measure restraint, eating concern, shape and weight concerns. The possible responses range from zero to three (0 = 0 days, 1 = 1–2 days, 2 = 3–5 days, 3 = 6–7 days). The last two questions ask the respondent to report their attitudes towards their weight over the past seven days. The responses are given in a Likert scale (0 = not at all, 1 = slightly, 2 = moderately, 3 = markedly). The total score is retrieved by summing up the responses. A higher total score represents a higher expression of ED.

## Results

### Data handling

#### Gaze cueing task

Errors were defined as trials in which participants responded too quickly (i.e., RTs < 100 ms, known as anticipations: 0.6% of trials), too slowly (i.e., RTs > 1000 ms, known as time outs: 0.7% of trials) or responded during a no-target trial (6% of trials, known as catch trials: 1.2% of trials), and were removed from analyses. Anyone with greater than 10% errors was also removed from the analyses, resulting in a loss of two participants (one control participant and one ED participant). Due to a technical error, data was not obtained for an additional control subject.

#### Video watching task

Dynamic interest areas (IAs) were drawn around the actors’ eyes, heads and the background using DataViewer software (SR Research; Mississauga, ON). Gaze behaviour was calculated by determining the proportion of time fixating on the three interest areas (IAs)—the eyes, heads and background—by adding up the total proportion of time fixating on each of the IAs across the entire film clip. Technical issues with the eye tracker resulted in the loss of one control participant.

#### Preferred interpersonal space task

Nose-to-nose distances were calculated between participants and confederate 3 times to obtain an average nose-to-nose distance. We did not obtain nose-to-nose measures for one ED and one DE participant, and only 31 control participants were included in these analyses due to one outlier.

### Data analyses

The data were analyzed in the following order. First, we analyzed whether performance varied across the control, DE, and ED groups for the three experimental tasks. In addition, we computed the scaled Jeffrey-Zellner-Siow (JZS) Bayes factor (BF) to determine whether our main findings were in favour of the null hypothesis using the default Cauchy r scaling value (0.707)^68^. This approach employs a noninformative prior, employs odds ratios rather than reference cutoffs, and takes into account sample size. Following this, we investigated whether our sociocognitive measures varied systematically with eating disorder symptomology as measured using the EDE-QS questionnaire^66^, applying Pearson correlation analyses.

#### Gaze cueing task

We ran three repeated-measures ANOVAs, one for each of the three groups (control, DE, ED), as a function of *Cue validity* (cued, uncued) and *Cue-target interval* (100, 400, 700 ms) as within-subject factors (see Fig. 1A for an example task sequence). In line with prior work^23,34,63^, and as depicted in Fig. 1B,C, the control group showed a typical gaze cueing effect, with faster responses for cued as compared to uncued trials [*Cue validity: F*(1,31) = 10.4, *p* < 0.01, η_p_^2^ = 0.25, 95% CI [397.6, 404.8]], that did not vary as a function of timing [*Cue validity* x *Cue-target interval*, *F* < 1, *p* > 0.4, , η_p_^2^ = 0.03]. In contrast, neither the DE nor the ED group showed significant gaze cueing effects [*Cue validity:* DE: *F*(1,17) = 1.1, *p* > 0.3, η_p_^2^ = 0.06, 95% CI [426.9, 430.0]; ED: *F*(1,17) = 1.7, *p* > 0.2, η_p_^2^ = 0.09, 95% CI [414.4, 417.7], nor did this change with target presentation timing [*Cue validity* x *Cue-target interval*, both *Fs* < 1, *ps* > 0.7, η_p_^2^s < 0.02], indicating no spontaneous attention shifts in response to viewing averted gaze.

We next calculated the magnitude of the gaze cueing effect (average uncued RT– average cued RT) for each participant, and conducted t-tests to determine whether the magnitude was significantly greater than zero for each group. Our control participants demonstrated a robust gaze cueing effect (t(31) = 3.2, p < 0.01, 95% CI [3.1, 13.8], scaled JZS BF = 12.54 favouring the alternative), while the DE and ED groups did not (DE: t(17) = 1.0, p > 0.3, 95% CI [-3.1, 9.2], scaled JZS BF = 2.60 favouring the null; ED: t(17) = 1.3, p > 0.2, 95% CI [-2.1, 8.8], scaled JZS BF = 2.0 favouring the null).

#### Video watching task

Based on prior work focusing on looking times for the eyes specifically^47,69^, we ran independent-samples t-tests to compare the proportion of time participants spent looking at the actors' eyes across the control, DE, and ED subgroups (see Table 2 for all data). Perhaps unexpectedly, the proportion of time controls spent looking at the eyes did not differ from either the DE group (*t*(48) = − 1.6, *p* = 0.1, Cohen’s d = 0.48, scaled JZS BF = 1.26 favouring the null) or the ED group (*t* < 1, *p* > 0.6, Cohen’s d = 0.15, scaled JZS BF = 3.08 favouring the null). The DE group looked significantly more at the eyes as compared to the ED group (*t*(34) = 2.1, *p* < 0.05, Cohen’s d = 0.69, scaled JZS BF = 1.60 favouring the alternative). In contrast to the gaze cueing task's findings, the passive video watching task appears sensitive to differences in gaze behaviour across the DE and ED groups.

**Table 2:** Table 2 depicts the average percentage of time (and corresponding standard deviation, SD) that each group spent fixating within each dynamic interest area region.

Group	Eyes (% ± SD)	Head (% ± SD)	Background (% ± SD)
Control	18.2 ± 11.5	54.3 ± 8.5	27.5 ± 18.2
DE	23.2 ± 8.7	55.9 ± 4.5	20.9 ± 12.3
ED	16.5 ± 10.7	51.5 ± 15.4	32.0 ± 24.2

#### Preferred interpersonal space task

**Figure 2:**
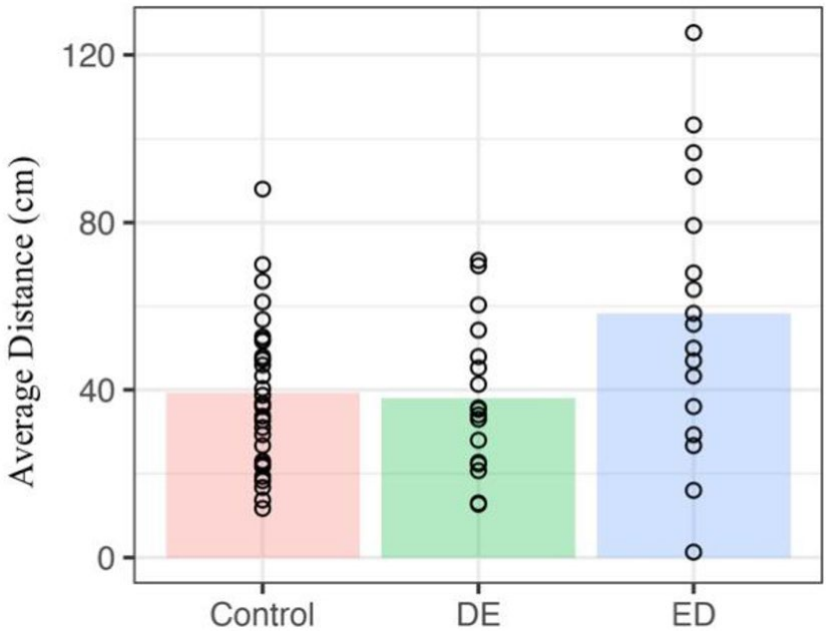
Depicts both the average nose-to-nose difference (in cm) as a function of group (coloured bars), along with the individual data points illustrating each participant’s preferred social distance.

On average, the preferred social distances were 39.3 cm for the control group, 38.1 cm for the DE group and 58.3 cm for the ED group (see Fig. 2). The numerical difference between the ED group and the other two groups were confirmed with independent samples t-tests, whereby the control group (*t*(46) = -2.60, *p* < 0.05, Cohen’s d = 0.72, scaled JZS BF = 4.08 favouring the alternative) and the DE group (*t*(32) = -2.22, *p* < 0.05, Cohen’s d = 0.76, scaled JZS BF = 2.05 favouring the alternative) had smaller preferred social distances as compared to the ED group (note, there was no difference between the control and DE group, *t*(46) < 1, *p* > 0.8, Cohen’s d = 0.07, scaled JZS BF = 3.29 favouring the null).

#### Correlation of eating disorder symptomology and social attention

**Figure 3:**
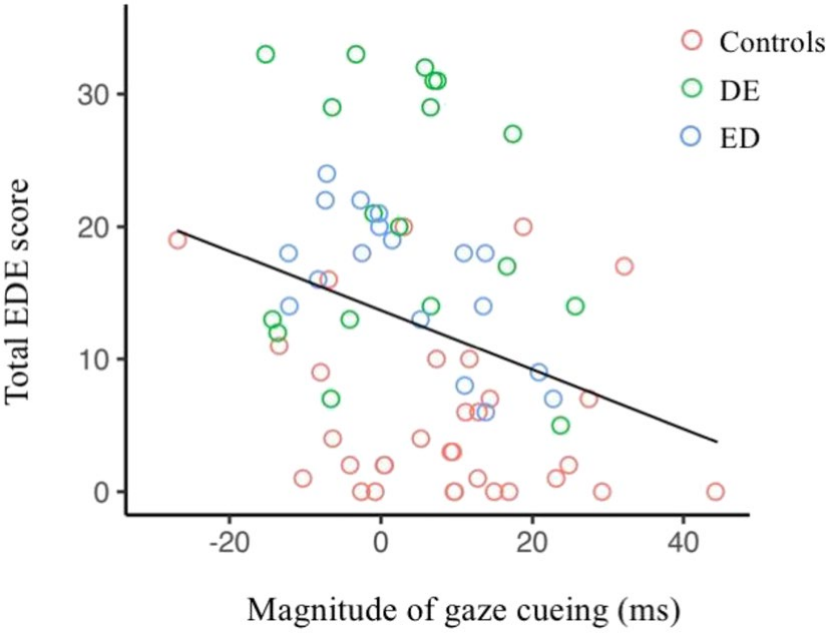
Depicts each participant's average magnitude of gaze cueing (x-axis) as a function of total EDE-QS score (y-axis). Different coloured data points denote group membership.

Finally, we investigated whether eating disorder symptomology varied systematically with our social attention measure obtained from the gaze cueing task. To do so, we calculated the magnitude of gaze cueing (see Fig. 1C) by calculating the average RT for cued and uncued trials and subtracting the cued from uncued RTs (i.e., average uncued RT—average cued RT); positive scores indicate the gaze cue has preferentially directed attention, and zero or negative scores indicate attention is not affected by gaze. We found a significant negative correlation between the total EDE-QS^66^ score and the magnitude of gaze cueing (r = − 0.32, p < 0.01; Fig. 3), with greater eating disorder symptom severity in the past week related to smaller magnitudes of gaze cueing. In other words, those individuals who reported more eating disorder symptoms were less likely to shift their attention in the direction gazed at by an isolated face.

## Discussion

The purpose of this study was to test the efficacy of various sociocognitive tasks at stratifying performance across a community sample of women either with or without a formal eating disorder diagnosis, along with controls. We found that women in both the ED and DE groups did not show a reliable magnitude of gaze cueing for the gaze cueing task, while control participants showed intact gaze cueing. Intriguingly, we found that reduced or reversed magnitudes of gaze cueing were linked to higher acute eating disorder symptomatology, as per the EDE-QS^66^. In the video watching task, we found a different pattern of results, whereby the DE group fixated significantly more on the actors’ eyes as compared to the ED group. In contrast, for the preferred interpersonal space task where participants were placed in a real-life, face-to-face interaction, the ED group had a significantly larger preferred interpersonal distance than the control and DE groups, who shared a similar preferred distance. Together, our work suggests the different tasks are not equally sensitive at detecting variations in social cognition, which we discuss further below.

Our results for the cueing task suggest that while the task is sensitive enough to detect the presence or absence of social attention for each group, the task was not able to distinguish between the two eating disorder groups. One possibility for this finding could be that the cueing task is simply not sensitive enough to detect differences between the ED and DE groups, while a second possibility could be that there are truly no significant differences between those with an official eating disorder diagnosis versus those who have disordered eating behaviours but do not have a formal diagnosis. These results dovetail with those from Dalmaso and colleagues^10^, who found that individuals with AN did not produce a gaze cueing effect, yet also extends this conclusion to a broader eating disorder population that spans specific diagnoses. What we mean by this is, aside from the current study, we are unaware of any other research differentiating between women who are officially diagnosed with an eating disorder and those who present with eating disorder symptomology without official diagnosis. More broadly, this finding provides evidence suggesting that spectrum-like features exist for eating disorder symptomatology and diagnoses.

The video watching task provided us with a naturalistic measure of overt attention, parsing fine-grained eye movement differences between the ED and DE groups. Following the cueing task results, we expected that the ED and DE groups would spend less time looking at the eyes during the video. However, we found that the DE group looked at the eyes significantly more than the ED group, with the proportion of time controls looked at the eyes falling between the two groups. While these results may seem surprising, we refer to prior work to contextualize our findings. Specifically, while the data show that some individuals with reduced sociocognitive functioning, such as those with AN and ASD, tend to spend *less* time looking at the eyes in static images^9,50^, other work demonstrates that abnormal attentional orienting can also consist of *excessive* fixations on social stimuli, as seen in Williams syndrome^52,70,71^. Thus adverse consequences can exist on each side of the spectrum; individuals who tend to focus less on faces may miss important social cues, while individuals who over-fixate on faces may appear to be threatening during social interactions^52^. Both patterns can lower social adjustment and impact social quality of life^72^. Of note, there has been very little work to date investigating passive looking rates to video clips in those with reduced social functioning, however with their increased ecological validity and natural social interactions, future work should tease apart potential differences between looking at static social scenes versus dynamic social interactions across individuals with different levels of social functioning.

The preferred interpersonal space task introduced a real-life component to our study to supplement the laboratory-based sociocognitive tasks. Our results show that the ED group preferred a larger distance between themselves and a confederate, as compared to the DE and control groups, who showed no significant differences in interpersonal space. Similar to the video-watching task, the preferred interpersonal space task also appears to function well enough to differentiate between our ED and DE groups, suggesting that this task may be a suitable tool when studying sociocognitive functioning. Prior work has investigated preferred interpersonal space in those with borderline personality disorder and social anxiety, and have found a clearcut difference between controls and these groups, where those diagnosed with borderline personality disorder or social anxiety have shown larger preferred social distances as compared to controls^4,5^. In contrast, our work, along with prior work investigating preferred social distance in those with ASD, has revealed nuances; preferred social distance in those with ASD seems to be affected more by level of anxiety, and our work suggests that only those with an official eating disorder diagnosis have a larger preferred social distance. We propose that this nuanced pattern of results could be indicative of a spectrum-like composition for individuals with an eating disorder, similar to the spectrum-like nature of ASD. Intriguingly, our results dovetail with recent work investigating inhibition of return (IOR) in those with and without an AN diagnosis^73^. IOR is a phenomenon whereby participants are slower to respond to targets that appear in the same location as the previous trial, suggesting that we inhibit our attention from returning to the same location over again^36^. Of note, when completing this task with another person, while control participants demonstrated inhibition effects for both their own previous responses as well as their partner’s (a so-called social IOR effect^74^) those with AN were found to *only* produce social IOR, which was larger in magnitude than controls^73^. When examined alongside our finding of greater preferred interpersonal space for the ED group, there is evidence to suggest that individuals with an eating disorder may be overly attentive to other individuals in real life social interactions, which manifests as providing additional space between themselves and others. Further, as the social IOR paradigm has good ecological validity, the convergence between findings from the stop-signal and sIOR tasks lends additional credibility to the stop-distance paradigm.

Taken together, our investigation demonstrates at least two benefits of using multiple sociocognitive measures with various levels of complexity to parse differences between highly diverse populations. The first insight of our work is that our study demonstrated that the way in which a construct is operationalized may fundamentally change the conclusions one can draw. Specifically, in our case, each task yielded a slightly different picture. For example, if only the gaze cueing task was used, then our data would have both complemented and extended those of Dalmaso and colleagues^10^; namely, the conclusion would be that social attention is altered not only for those with anorexia nervosa, but also in a community sample of those with and without an official eating disorder diagnosis. While this would be an important extension of prior work, the conclusion would also potentially erroneously suggest that there is no difference in social attention across those with an eating disorder versus those with disordered eating. In contrast, the additional eye tracking and preferred social distance tasks provided a different data pattern, with greater proportions of time looking towards the eyes for those presenting with DE, and larger real life preferred social distances for those in our ED group. When examining sociocognitive functioning through such a holistic lens, we arrive at different conclusions—one that demonstrates a potential spectrum of eating disorder behaviours that might not necessarily be captured by one single task alone. Therefore, there is strong support for conducting research that assesses multiple measures of a construct within the same group of individuals.

The second important insight highlights the validity of using community samples to study social cognition in those with an eating disorder. While prior work has adopted the approach of focusing on homogeneous groups^9–11^, which has clear methodological benefits, we aimed to better understand whether various facets of social cognition do or do not differ between groups *as well as* within individuals with different eating disorder presentations. Our study found some evidence of reduced sociocognitive functioning in both the ED and DE groups, suggesting that changes in social cognition may be further-reaching than prior research suggests. This could be due to the fact that the prevalence of eating disorder symptoms found within a clinical sample varies from that of larger community sample, with greater instances of purging in bulimia nervosa, and binge/purge-type AN in community samples^75–77^. By virtue of recruiting participants from a clinical sample, only specific symptomatic dimensions may be present, rather than the array of symptoms found across those who exhibit disordered eating behaviours^78^. Thus it could be that the heterogenous constellation of symptoms in our eating disorder groups explain the pattern of results seen in this study. While there is no prior research that can directly support our interpretations, the broader claim holds, namely that individuals with diverse disordered eating profiles, officially diagnosed or not, appear to have difficulties with aspects of social cognition, highlighting the importance of looking beyond key symptoms addressed within a clinical sample.

No study is without limitations, including the current investigation. For instance, by its very nature, a diverse group will be comprised of smaller sub-diagnostic groups, which may have limited the study’s power. However, a secondary goal was to investigate whether there is variability in social cognition across different instantiations of eating disorder symptomology, thus requiring a diverse sample. A second limitation was the self-report nature of one’s diagnosis (formal or informal). While this is a legitimate concern, in spite of participant self-report we found differences in social cognition as a function of category membership, supporting the notion that the difficulties with sociocognitive function highlighted in those with AN extend to others who exhibit disordered eating behaviours.

In sum, we believe that multiple experimental measures, varying in complexity, are necessary to draw conclusions around particular constructs, especially when the investigation involves heterogeneous community samples. The current study adopted this approach and found that two sociocognitive tasks were efficacious in parsing between ED and DE performance, while the third was able to make a broad-stroked distinction across controls and those with ED or DE. By combining multiple tasks, cross-task evaluations about efficacy and sensitivity of the tasks themselves can be made. Thus, we argue for the importance of drawing from diverse community samples and using various tasks. At the same time, we recognize that this approach may lead to less clear-cut patterns of results. In spite of potential limitations, we believe that multi-task experiments offer unique insights about sociocognitive measuring in the lab, are more representative of real-life behaviours, and underscore the human mind's impressive complexities.
